# Molecular characterization of human adenoviruses associated with pediatric respiratory infections in Karachi, Pakistan

**DOI:** 10.1186/s12879-024-09415-9

**Published:** 2024-05-29

**Authors:** Khalid Mahmood, Waqar Ahmed, Saba Farooq, Gul Habib, Muhammad Ashfaq Sindhu, Afshan Asif, Thomas Iftner

**Affiliations:** 1 National Institute of Virology, Dr. Panjwani Center for Molecular Medicine and Drug Research, International Center for Chemical and Biological Sciences, University of Karachi, Karachi, 75270 Pakistan; 2 Department of Microbiology, Abbottabad University of Science and Technology, Havelian, Abbottabad, 22010 Pakistan; 3 Department of Pediatrics, National Institute of Child Health, Jinnah Sindh Medical University, Karachi, Pakistan; 4 Department of Pediatrics, Sindh Government Children Hospital, Karachi, Pakistan; 5 Institute of Medical Virology and Epidemiology of Viral Diseases, University Hospital Tuebingen, Elfriede-Aulhorn-Str. 6, 72076 Tubingen, Germany

**Keywords:** Adenoviruses, Pediatric, Hexon, Respiratory tract infections, Karachi

## Abstract

Human adenoviruses (HAdVs) are a diverse group of viruses associated with respiratory infections in humans worldwide. However, there is a lack of research on the genetic diversity and epidemiology of HAdVs in Pakistan. This study characterized HAdVs in pediatric patients with respiratory tract infections in Karachi, Pakistan, between 2022 and 2023. We analyzed 762 nasopharyngeal samples of children ≤ 5 years. DNA extraction, followed by PCR targeting E2B and hexon genes, was carried out. Data analysis was performed on SPSS 25.0, and phylogenetic analysis of hexon gene was performed on MEGA 11. HAdV was detected in 7.34% (56/762) of patients round the year, but at a significantly higher rate during the winter season. Age was insignificantly associated with HAdV incidence (*p* = 0.662), but more than 62.5% (35/56) of positive cases were younger than 10 months. The circulating HAdVs were identified as six different types from species B (78.57%) and C (21.42%), with the majority of isolates found to be like B3. HAdV was found to be co-infected with bocavirus (5.4%) and measles (7.14%). These findings revealed a high frequency and genetic diversity of respiratory HAdVs in Karachi, Pakistan. We conclude that periodic and continuous surveillance of adenoviruses and other respiratory pathogens is necessary to improve the prognosis and management of respiratory diseases, thereby reducing the child mortality rate in Pakistan.

## Introduction

Human adenoviruses (HAdVs), classified as non-enveloped double-stranded DNA viruses in the Mastadenovirus genus of the Adenoviridae family, are 70–100 nm in diameter with an icosahedral shape and a linear DNA of about 36 kb (Shieh 2022; Wang et al. 2021). Depending on the haemagglutination reaction, fiber gene length and GC content of their genome, 113 types of HAdVs have been identified and classified within seven species, A-G (Wu et al. 2022). HAdVs can be transmitted through respiratory droplets, direct contact with contaminated surfaces, or fecal-oral route with an incubation period of 2 to 14 days (Ghebremedhin 2014; Xu et al. 2022). HAdVs are major public health concerns and are linked to various clinical manifestations of multiple mucosal sites, including respiratory, gastroenteritis, ocular and urinary tract (Das and Basu 2020; Grand 2023; Nanmoku et al. 2016). They are associated with chronic infections in both healthy and immunocompromised patients and can produce severe or deadly illnesses in children and the elderly (Spaeder et al. 2022; Zhao et al. 2021).

HAdVs contribute for around 1–7% of respiratory tract infections in adults and 5–10% in children, resulting in pneumonia in 20% of infants and newborns worldwide (Jabeen et al. 2021; Spaeder et al. 2022; Wang et al. 2021). Previous studies show that subjected to the demography and epidemiological settings, specific serotypes of the HAdVs are responsible for particular clinical manifestations and disease progression. Species of HAdV-B, C, and E are majorly involved in causing respiratory infections, mostly asymptomatic, mild and self-limiting. However, HAdV-B3, HAdV-B7, HAdV-E4 and a recombinant virus type of HAdV-B3/B7 have been involved in often severe infections and outbreaks in military recruits and pediatric populations and immunocompromised individuals (Coleman et al. 2021; Majumdar et al. 2023).

In Pakistan, acute respiratory tract infections (ARIs) account for 20–30% of all child deaths and are the leading cause of hospitalization (Naz et al. 2019). Approximately 71,000 children die each year in Pakistan as a result of pneumonia (Sajed 2019). Nearly 50% of children in Sindh missed vaccinations during the COVID-19 pandemic, leading to over 10,000 reported cases of measles (Naeem et al. 2022). It increased the vulnerability of the population to several diseases, particularly the viral infections, underlining the significance of baseline surveillance and genetic study into endemic viruses circulating within the region. HAdVs have been studied extensively for more than 50 years, still there is no information on the prevalence and circulating genotypes of HAdVs in Pakistan. Karachi is one of the largest city of Pakistan and accommodates over 20.0 million of the country’s population. The city is the country’s most linguistically, ethnically, socially, and religiously diverse metropolis (https://www.pbs.gov.pk/, 2023). Therefore, molecular characterization of HAdVs in Karachi would provide a scientific basis for the control and prevention of HAdV infections, as well as provide information on adenoviral strains for deployment of adenovirus-based vaccinations in Pakistan. In this study, we performed the molecular typing of respiratory adenoviruses detected during 2022–2023 in Karachi.

## Materials and methods

### Study design, ethical approval, and clinical specimen

We conducted a cross-sectional, retrospective study between May 2022 and September 2023 at the National Institute of Virology, Dr. Panjwani Center for Molecular Medicine and Drug Research, university of Karachi. The participating hospitals (Sindh Government Children Hospital, National Institute of Child Health, and Civil Hospital) and institutional independent ethics committees approved the study (ICCBS/IEC-078-NS-2022/Protocol/1.0. A sample size of 601 was estimated using the Social Science Statistics online tool, with a 95% confidence level, a margin of error of ± 4, and 0.5 estimated proportion. We adopted a purposive sampling strategy, included 762 nasopharyngeal swab (NPS) samples from children aged between ≥ 01 months to ≤ 5 years, who hospitalized or visited emergency department with respiratory infections. Guardians of children were fully informed about the HAdVs epidemiological surveillance and written consent was obtained before they were included in the study. NPS samples were placed in chilled viral transport medium at the collection site, transported to the laboratory, and stored at -80℃ before use. Demographic information was obtained from guardians while clinical information on patient was collected from the on-duty medical officer. Data on meteorological factors, such as temperature (℃), humidity (%), wind speed (mph), and rainfall (mm) was obtained by accessing https://weatherspark.com/countries/PK (https://weatherspark.com).

### Extraction of nucleic acid and real time PCR detection of HAdVs

Qiagen’s *QIAamp DNA Mini Kit (Cat# 51,306, Germany)* was used to extract nucleic acid from 140 µL of clinical sample in 60 µL elution buffer following the manufacturer’s instructions. The detection of HAdVs was performed using *GeneProof Adenovirus PCR Kit (Cat# ADV/ISEX/100, Czech Republic)*. Briefly, 10 µL of sample or calibrator (serial dilutions of positive control provided by the kit) was added to 30 µL of the reaction mixture. PCR was performed at thermal profile: decontamination at 37 °C for 2 min, initial denaturation at 95 °C for 10 min and 45 cycles of denaturation at 95 °C for 5 s (s), annealing at 60 °C for 40 s, extension at 72 °C for 20 s. Positives samples were screened based on Cq values ≤ 38.

### Nested PCR and sequencing

Nested PCR targeting the hexon gene was performed using absolute mastermix *Molequle-On (Cat# AM-M-001-1250, New Zealand)*. The outer primers used were Forward (5′-caggatgcttcggagtacctgag-3′), and Reverse (5′-tttctgaagttccactcgtaggtgta-3′), while the inner primers were Forward (5′-gtaggcgacaacagagtgct-3′), and Reverse (5′-ctgcaacagcatccctacca-3′) (Wu et al. 2022). The thermal profiling procedure we used for both rounds of PCR was the same, starting with an initial denaturation at 94 °C for 5 min, 40 cycles of denaturation at 94 °C for 30 s, annealing at 58 °C for 1 min, extension at 72 °C for 2 min, and 1 cycle of final extension at 72 °C for 10 min. Visualization of amplified product (1685 bp and 553 bp) was carried out in 2% TAE (Tris-acetate-EDTA) agarose gel. The targeted region of the gel was excised and purified using a *MQ PCR Product Purification Kit (Cat# PPK-M-002-100, New Zealand)*. Twenty eight positive samples were sequenced using forward primer of the hexon gene from macrogen’s commercially available services.

### Bioinformatics analysis

All the sequences were subjected to Basic Local Alignment Search Tool (BLAST) to find homologous nucleotide sequences from gene bank. 26 HAdVs reference sequences that were closely related to sequenced hexon sequences were retrieved and used for phylogenetic analysis. The Clustal W method was used for sequence alignment, and the maximum Likelihood approach and Tamura-Nei model were used to create the phylogenetic tree on Mega 11 software. The accuracy of the tree was assessed by using 1000 bootstrap repetitions.

### Statistical analysis

The statistical analysis was performed using the *Statistical Package for Social Sciences (SPSS) version 25.0* on the demographic and clinical data. The statistical significance (*p* ≤ 0.05) of epidemiological data was determined by Chi-square. The Prism 9 software was used to make graphs.

## Results

### Demographic and clinical characteristics of the target population

The study, which lasted 15 months from May 2022 to September 2023, examined 762 nasopharyngeal samples taken from three different hospitals in Karachi. There were 458 males (60.1%) and 304 females (39.9%). The age of children varied from > 01 month - ≤ 05 year. They were divided into three groups: > 01 month - ≤ 01 year, ≥ 01 - ≤ 02 years, and ≥ 02 - ≤ 05 years. The average and median ages were 1.14 and 0.67 years, respectively. More than half of children (51.7%) were eight months or younger (Table 1).

Three distinct sections from each hospital contributed differently to the sampling: the high care unit for pulmonary disease has highest contribution 78.5%, followed by the intensive care unit 19.1%, and the emergency department 2.4%. Upper respiratory tract infections (UTIs) were diagnosed in 24.4% (186/762), while lower respiratory infections (LTIs) were found in 60.2% (459/762), with pneumonia and bronchiolitis accounting for 57.1% (435/762), and 3.1% (24/762) respectively. A large number of pneumonia patients (13.1%, 100/762) had comorbidities such as measles, sepsis, Down syndrome, Cushing syndrome, and cerebral palsy.

We analyzed clinical manifestations of HAdV positive patients and found that 98.2% had a cough, whereas 80.4%, 75.0%, 71.4%, and 42.9% experienced difficulty in sleeping, chest congestion, fever, and conjunctivitis respectively. Patients length of stay at hospital, family history of lung diseases and smoking and coinfection with bocavirus and measles were also analyzed (Table 2).

**Table 1:** Characteristics of gender and age of the HAdV positive patients in karachi, pakistan, July 2022 – September 2023

Variable	Total cases (*N*)	HAdV positive (*N*)	HAdV positive (%)	*P* value
**Gender**				
Male	458	26	5.7%	0.297
Female	304	30	9.9%	
**Age**				
> 01 month - ≤ 01 year	493	39	7.9%	0.662
≥ 01 - ≤ 02 years	119	7	5.9%	
≥ 02 - ≤ 05 years	101	10	9.9%	

### Epidemiology of HAdVs

HAdV was detected in 7.34% of pediatric patients with respiratory infections. Data showed that females had more HAdV infections (9.86%) than male (5.67%), but the difference was statistically not significant (Χ² = 1.087, *p* = 0.297). The prevalence of HAdV infections varied depending on the age group but not significantly (Χ² = 0.825, *p* = 0.662). It was highest in ≥ 02 - ≤ 05 years age group (9.9%) followed by > 01 month - ≤ 01 year (7.9%), and ≥ 01 - ≤ 02 years age group (5.9%).

**Table 2:** Clinical features of HAdV positive patients in Karachi, Pakistan, July 2022 – September 2023

	HAdV (*N* = 56)		HAdV (%)			*p* value
**Clinical Manifestations**						
Cough	55			98.2		0.043
Chest Congestion	53			94.6		
Sleeping Difficulty	45			80.4		
Fever	42			75		
Wheezing	40			71.4		
Conjunctivitis	24			42.9		
**Diagnostic Tests (CBC and CXR)**						
Pneumonia	33			58.9		0.046
Bronchiolitis	2			3.6		
URTIs	14			25		
Others	7			12.5		
**Length of Hospital Stay**						
No Hospitalization	38			67.9		0.047
1–3 Days	9			16.1		
3–7 Days	6			10.7		
> 7 Days	3			5.4		
**Family History of Lung Diseases**						
Lung diseases	21			37.5		0.054
Smoking	12			21.4		
**Co-infections**						
Human Bocavirus	2			3.6		0.053
Measles	4			7.1		

### Meteorological factors affecting viral seasonality during the study

**Fig. 1:**
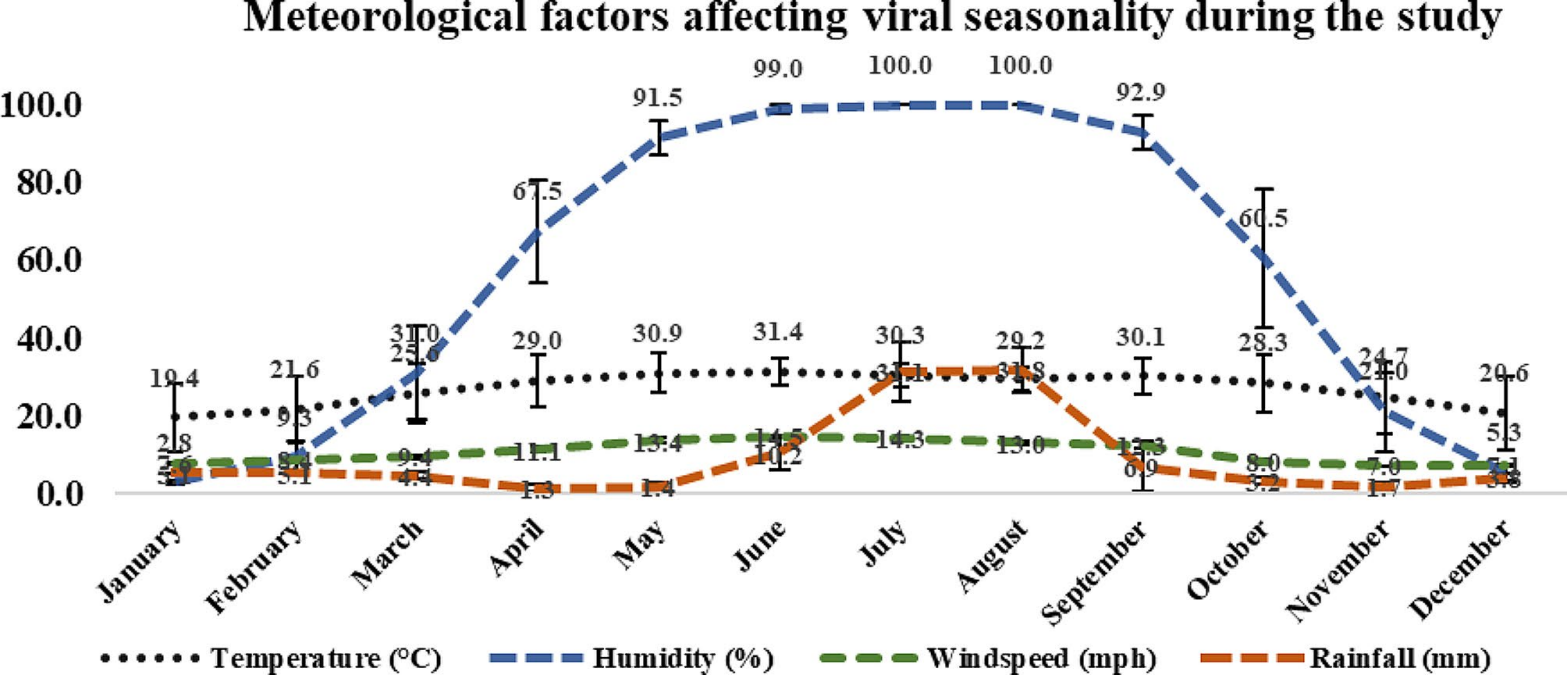
Meteorological factors: temperature (℃), humidity (%), wind speed (mph), and rainfall (mm) affecting viral seasonality during the study. Values are shown as means ± S.D

Several meteorological factors influence viral seasonality. We analyzed temperature (℃), humidity (%), wind speed (mph), and rainfall (mm) during the study. Data revealed that June was the hottest month and January was the coolest, with average temperatures of 31.4 and 19.4 ℃, respectively. July and August experienced the highest humidity (100%) with average rainfall of 31.1 and 31.8 mm, respectively. The fastest wind speeds were recorded in June and July, with an average speed of 23 mph (Fig. 1).

### Seasonal distribution of HAdVs

**Fig. 2:**
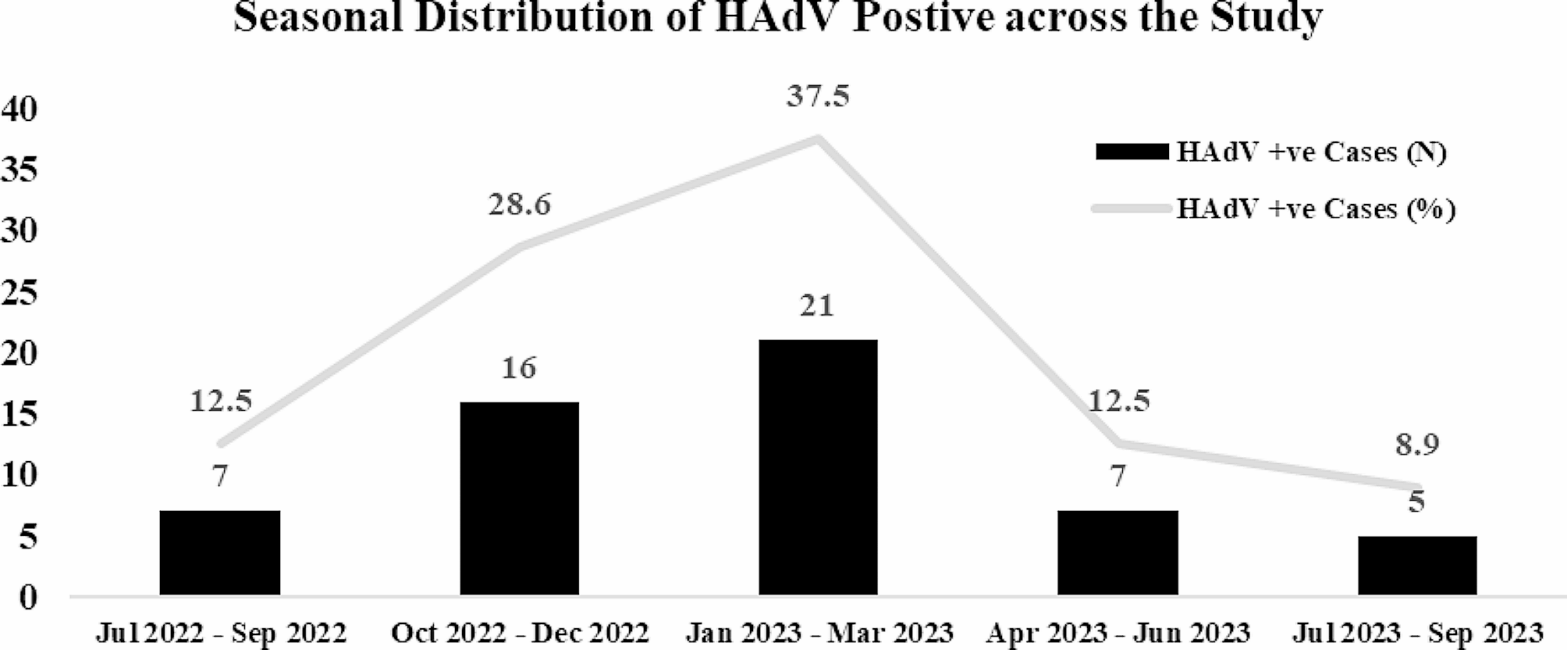
Seasonal distribution of HAdV positive cases over the duration of the study. The highest positivity rate of HAdV was found between January and March 2023

The Prevalence of HAdV infections also varied significantly throughout the year (*p* = 0.04). The infection rate peaked from January to March (37.5%) and then from October to December (28.1%). Between July and September, there was a considerable decline (10.7%) (Fig. 2).

### HAdV phylogenetic analysis and genotyping

**Fig. 3:**
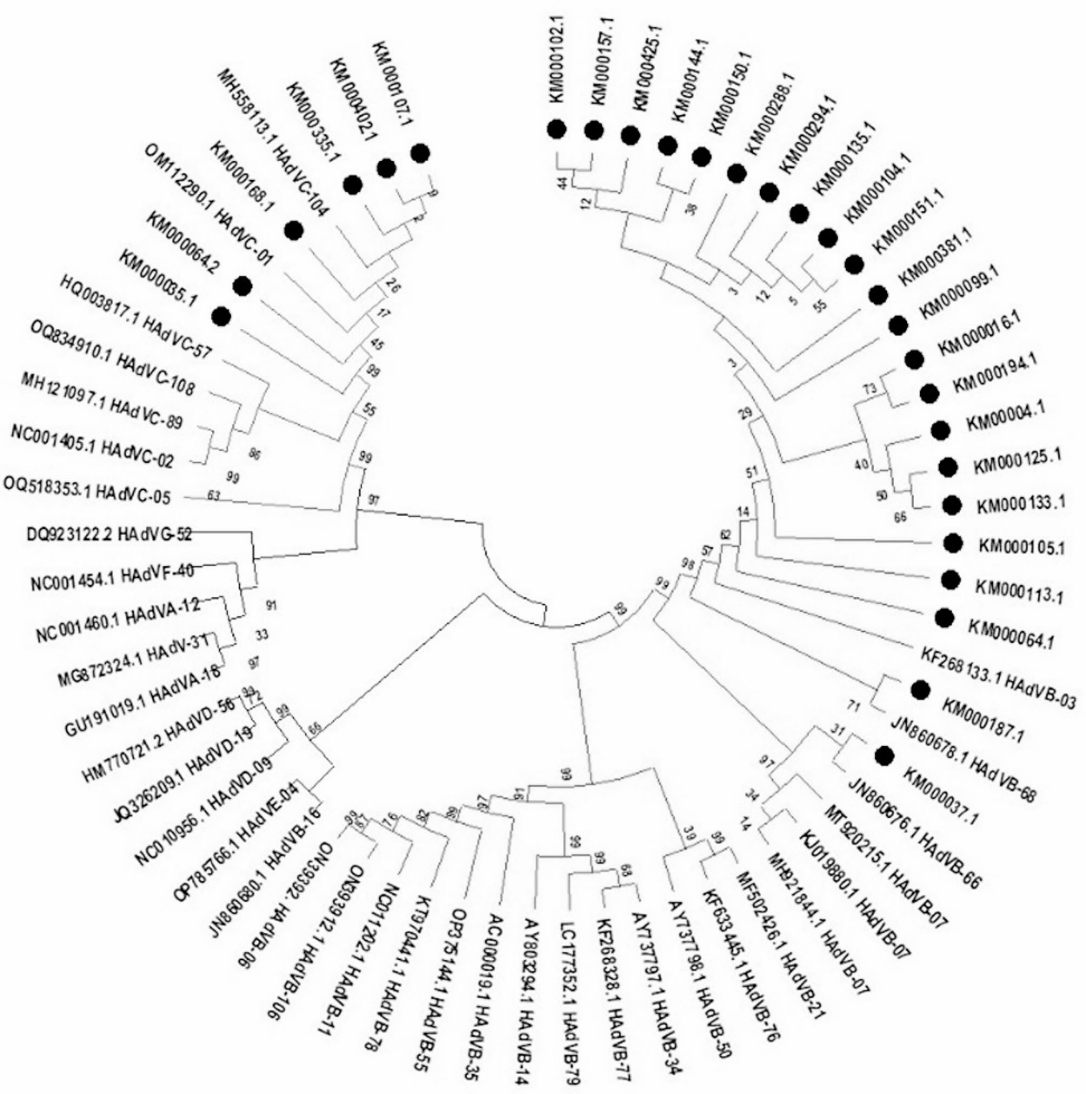
Evolutionary relationships of Texa. The phylogenetic analysis was built using the Maximum Likelihood approach and Kimura 2-parameter model (Kimura 1980). The tree with the highest log probability (-13424.53) is shown. The tree was constructed using MEGA software 11.0 with a 900-bp nucleotide sequence from the hexon gene using the neighbor-joining method. (Tamura et al. 2021). Red and blue colors reflect HAdV-B and C species respectively, with black dots marked represent samples sequenced in this study

This study considered all positive samples of HAdVs for genotyping, but following nested PCR, 28 (50.0%) samples showed effective amplification of the hexon gene of the specified size (1685 and 553 bp). The sequenced product has a Cq value between 15 and 29. The phylogenetic analysis revealed that HAdV B (HAdV-B3, HAdV-B66, and HAdV-B68) accounted for 78.57% cases, whereas C (HAdV-C01, HAdV-C05, and HAdV-C104) accounted for 21.42% cases (Table 3). These results showed that species B and C circulated simultaneously in Karachi, Pakistan and that HAdV-B3 like was the most prevalent genotype, followed by HAdV-C104 (Fig. 3).

**Table 3:** HAdV species and types (n) identified by partial hexon gene sequencing of positive cases (N)

Species (%, *n*/*N*)	Type	Number (%), *N* = 28
**B** (78.57%, 22/28)	3	20 (71.42%)
	66	1 (3.57%)
	68	1 (3.57%)
**C** (21.42%, 06/28)	1	1 (3.57%)
	5	2 (7.14%)
	104	3 (10.71%)

## Discussion

Acute respiratory tract infections are a leading global cause of mortality and morbidity. HAdV is a major pathogen in pediatric patients with ARIs. Several genetic variants of HAdV may exist within a single genotype, underlining the significance of frequent and detailed molecular characterization of HAdV to identify the prevalence of circulating genetic variants. The screening of HAdV does not exist in Pakistan due to poor health care facilities and patients are treated primarily on clinical presentation. This study was conducted on children suffering with ARIs who visited the emergency department or were admitted to three different hospitals in Karachi from July 2022 to September 2023. Our findings revealed 7.34% prevalence of the HAdV in Karachi in pediatric patients. It showed that females are at a greater risk of HAdV infections (9.86%) than male (5.67%) but not significantly. The rate of the virus infection also varied with age, being the

highest in ≥ 2 - ≤ 5 years (9.9%), and the lowest ≥ 1 - ≤ 2 years (5.9%). We observed that the frequency of HAdV varied significantly depending on the season. HAdV was also found to be co-infected with bocavirus (5.4%) and measles (7.14%).

The prevalence of HAdV vary across studies. We found a 7.34% prevalence, which is close to the 6.4% reported recently in Pakistan by Ahmed et al. (Ahmad et al. 2023). HAdV prevalence was found to be 5.64% (Yao et al. 2019), 7.08% (Xu et al. 2022), 9.4% (Xie et al. 2019), 5.8% (T. Liu et al. 2015a), 9.8% (Ukuli et al. 2023), and 3.8% (Wang et al. 2021). The disparity among these studies in HAdV detection rates might be caused by methodological variations, the size and length of the research, the time period during which the sample was obtained, and immunological status of the patients.

Age of the patient influences HAdV infections. Studies have indicated that children between the ages of six months and 5 years are more susceptible to HAdV than older (Liu et al. 2015b), albeit this varies among these age groups (Hong et al. 2001; Yao et al. 2019). Similarly, Wang et al. 2021 observed that HAdV positivity was highest (80%) in children under the age of five (Wang et al. 2021). In line with these findings, we noticed that the incidence of HAdV varied significantly among the various age groups studied (Table 1). The highest frequency in the age range ≥ 2 - ≤ 5 years (9.9%), may be related to increasing contact among kindergarten children, and poor hygiene practices.

Most viruses show certain seasonal variability. Price et al., investigated the impact of different weather conditions on respiratory viruses seasonality over a period of six-years and showed the prevalence of HAdV was to be year-round, but it surged during the cool season, favoring temperatures around 9 °C (Du Prel et al. 2009; Price et al. 2019). In agreement with these findings, we also demonstrate that HAdV infections persist throughout the year, with the highest number arising during the cold season. Other studies also documented similar trend of weather effect on the incidence of HAdV infections (Li et al. 2015; Rosete et al. 2008). These findings, however, contrast those of Yao et al., who found a significant incidence in the summer. The disparity in these observations might be due to differences in geographic location, length of sunlight, and other environmental factors.

In this study, six different types of HAdVs were identified, representing HAdV B and C: HAdV B3, B7, B66 and B68 HAdV C1, C5, and C104. The prevalence of HAdV B3 was highest among different types of HAdVs, which is in agreement with previous studies that showed severe and widespread occurrence of infections caused by B and their association with outbreaks (Coleman et al. 2020; Wang et al. 2023). The high prevalence and sporadic outbreaks of HAdV-B3 can be linked to the great diversity in the antigenic part of the hexon protein (Haque et al. 2018). This study, for the first time characterizes the different types of adenovirus in pediatric patients in Karachi, Pakistan. The strength of the study was the inclusion of patients from three main hospital settings in the city, allowing for a diverse geographical distribution. Our study demonstrates that immunocompromised youngsters are more prone to get adenoviral illness while in the hospital. Our study has certain limitations: it is based on small sample size and a narrow range of age. Moreover, only 28 partial sequences of the hexon gene were sequenced. Therefore, a study with large sample size, broader age range, and comprehensive genome analysis to determine the level of genetic variation and probable recombination that occurred in the strains is necessary to verify our results. We also strongly recommend periodic and constant genotype surveillance of HAdV in order to make informed decisions about treatment and control strategies.

## Data Availability

Accession numbers and links for sequences analyzed in this work are available in the Gene Bank: OR567870: https://www.ncbi.nlm.nih.gov/nuccore/OR567870 PP196670: https://www.ncbi.nlm.nih.gov/nuccore/PP196670 PP196671: https://www.ncbi.nlm.nih.gov/nuccore/PP196671 PP196672: https://www.ncbi.nlm.nih.gov/nuccore/PP196672 PP196673: https://www.ncbi.nlm.nih.gov/nuccore/PP196673 PP216193: https://www.ncbi.nlm.nih.gov/nuccore/PP216193 PP216202: https://www.ncbi.nlm.nih.gov/nuccore/PP216202 PP216203: https://www.ncbi.nlm.nih.gov/nuccore/PP216203 PP216211: https://www.ncbi.nlm.nih.gov/nuccore/PP216211 PP216213: https://www.ncbi.nlm.nih.gov/nuccore/PP216213

## Abbreviations

ARIs: Acute respiratory tract infections
BLAST: Basic Local Alignment Search Tool
bp: Base pair
℃: Degrees Celsius
DNA: Deoxyribonucleic acid
HAdV/s: Human adenovirus/es
LTIs: Lower respiratory infections
NPS: Nasopharyngeal swab
PCR: Polymerase chain reaction
SPSS: Statistical Package for Social Sciences
TAE: Tris-acetate-EDTA
UTIs: Upper respiratory tract infections
